# Genome Sequences and Comparative Analysis of Two Extended-Spectrum Extensively-Drug Resistant *Mycobacterium tuberculosis* Strains

**DOI:** 10.3389/fphar.2018.01492

**Published:** 2018-12-18

**Authors:** Masood R. Kayani, Yong-Chang Zheng, Fu-Cun Xie, Kai Kang, Han-Yu Li, Hai-Tao Zhao

**Affiliations:** ^1^School of Life Sciences, Tsinghua University, Beijing, China; ^2^Department of Liver Surgery, Peking Union Medical College Hospital, Chinese Academy of Medical Sciences and Peking Union Medical College, Beijing, China

**Keywords:** *Mycobacterium tuberculosis*, drug resistance, genome sequence, pangenome, virulence factor

## Introduction

Tuberculosis (TB) is a disease which has resulted in the deaths of ~one billion people during the last 200 years (Paulson, 2013). TB is typically caused by *Mycobacterium tuberculosis* and treated using various first- and second-line drugs which rapidly kill the bacteria in the initial phase and later clear the body of any remaining pathogenic cells. Despite the efforts, a recent report published by the world health organization (WHO) suggested that the pathogen is still causing ~10.4 million new infections and 1.7 million annual deaths. One of the greatest threats to the treatment of TB is posed by increased drug resistance in *M. tuberculosis*. For example, multidrug resistant (MDR) TB, which indicates resistance to rifampicin and isoniazid. It is estimated that only a small fraction of MDR-TB cases i.e., 7% are adequately diagnosed and appropriately treated (Toosky and Javid, 2014). Further aggravation of such cases, by added resistance to quinolones and injectable drugs, is termed as extensively-drug resistant (XDR) TB.

During last 10 years, more severe forms of drug resistant TB have also emerged in Italy, Iran, India and South Africa which are resistant to almost all first- and second- line agents (Migliori et al., 2007; Velayati et al., 2009; Udwadia et al., 2012; Klopper et al., 2013). These cases number 30 in total, which most probably is an under-estimate of the cases due to resource-limiting settings where tuberculosis is endemic. Two such cases were recently identified in Beijing, which showed phenotypic drug resistance against an extended panel of tested antibiotics (Li et al., 2015). Hence they were referred as the extended-spectrum extensively-drug resistant tuberculosis (XXDR) strains. One of the strains (Strain EF) was collected from a 64-year old HIV-negative patient who suffered type II diabetes and chronic hepatitis C. The second strain (Strain ZX) was collected from a 30-year old HIV-negative patient who was non diabetic and also showed negative outcomes for Hepatitis B and C viral testing. For both of these strains, the WGS analysis failed in the identification of causative mutations that rendered resistance against several antibiotics thus showed discordance with the phenotypic testing (Li et al., 2015). In this study, we assembled the genomes of these two strains and also performed their functional annotations. Our results can be key in development of new strategies for early diagnosis and better treatment of XXDR TB cases.

## Materials and Methods

### XXDR Strains

Two XXDR cases, which showed phenotypic resistance against an extended panel of tested antibiotics, have been previously reported. The complete description of these patients, their history, follow-up and the drug susceptibility testing (DST) profiles can be found in Li et al. (2015). The raw sequencing reads were retrieved from NCBI SRA database using accession number SRP058024 and processed for removal of adapters and sequences of quality lower than Q20 and any ambiguous bases (N).

### Genome Assembly and Annotation

The assembly of XXDR genomes was performed using SPAdes (Bankevich et al., 2012). The draft genome completion and contamination levels were assessed using CheckM v0.9.7 (Parks et al., 2015) whereas the contig order was corrected using Mauve (Darling et al., 2004) with *M. tuberculosis* H37Rv genome as a reference (accession number NC_000962.3). The draft genome sequences for the two XXDR strains were annotated using Prokka (Seemann, 2014) and RAST server (Aziz et al., 2008). Furthermore, the predicted proteins for the two genomes were also annotated for the identification of clusters of orthologous groups (COG), proteins families (Pfam) and TIGR families (TIGRfam). For the identification of CRISPRs, we used the webserver of CRISPRFinder.

### Comparative Genomic and Phylogenetic Analysis

For comparative genomic analysis, we downloaded 145 complete genome sequences for *M. tuberculosis* strains from the RefSeq database. All of the 145 Refseq genomes were re-annotated using Prokka for unbiased comparisons with the newly assembled XXDR genomes. From these, we chose 13 *M. tuberculosis* strains of different origin for phylogenetic analysis and calculation of average nucleotide identity (ANI) (Supplementary Table 1). The select 13 whole genomes were aligned using progressiveMAUVE algorithm and used for building the phylogenetic tree. ANI was calculated using JSpeciesWS (Richter et al., 2015) and visualized using R package (pheatmap). The complete list of reference genomes is provided in Supplementary Table 2.

### Pan-Genome Analysis and Identification of Intergenic Regions

The pan-genome of 145 *M. tuberculosis* reference genomes and XXDR strains was analyzed with Roary (Page et al., 2015). Few parameters were modified to produce alignments using MAFFT (Katoh et al., 2002) and to lower the amino acid identity value to 90% from the default value of 95%. Alignments for the core genes and accessory genes, generated by Roary, were used for generating phylogenetic tree using RAxML (Stamatakis, 2014). The tree files were visualized using the interactive tree of life (iTOL v4) webserver. For the detection of highly divergent intergenic regions (IGRs), we used Piggy (Thorpe et al., 2018) using the output from Roary.

### Antibiotic Resistance and Virulence Factor Identification

Antibiotic resistance (AR) mutations in the XXDR strains were identified using the resistance finder (ResFinder v3.0) tool (Zankari et al., 2012). To identify the virulence factors (VF), we aligned the predicted proteins from both genomes against the virulence factor database (VFDB) using blastp.

### Data Availability

The assembled genomes have been deposited at GenBank under the accession QGKJ00000000 and QGKK00000000. The versions described in this paper are QGKJ01000000 and QGKK01000000.

## Results

### General Features of the XXDR Genomes

**Figure 1:**
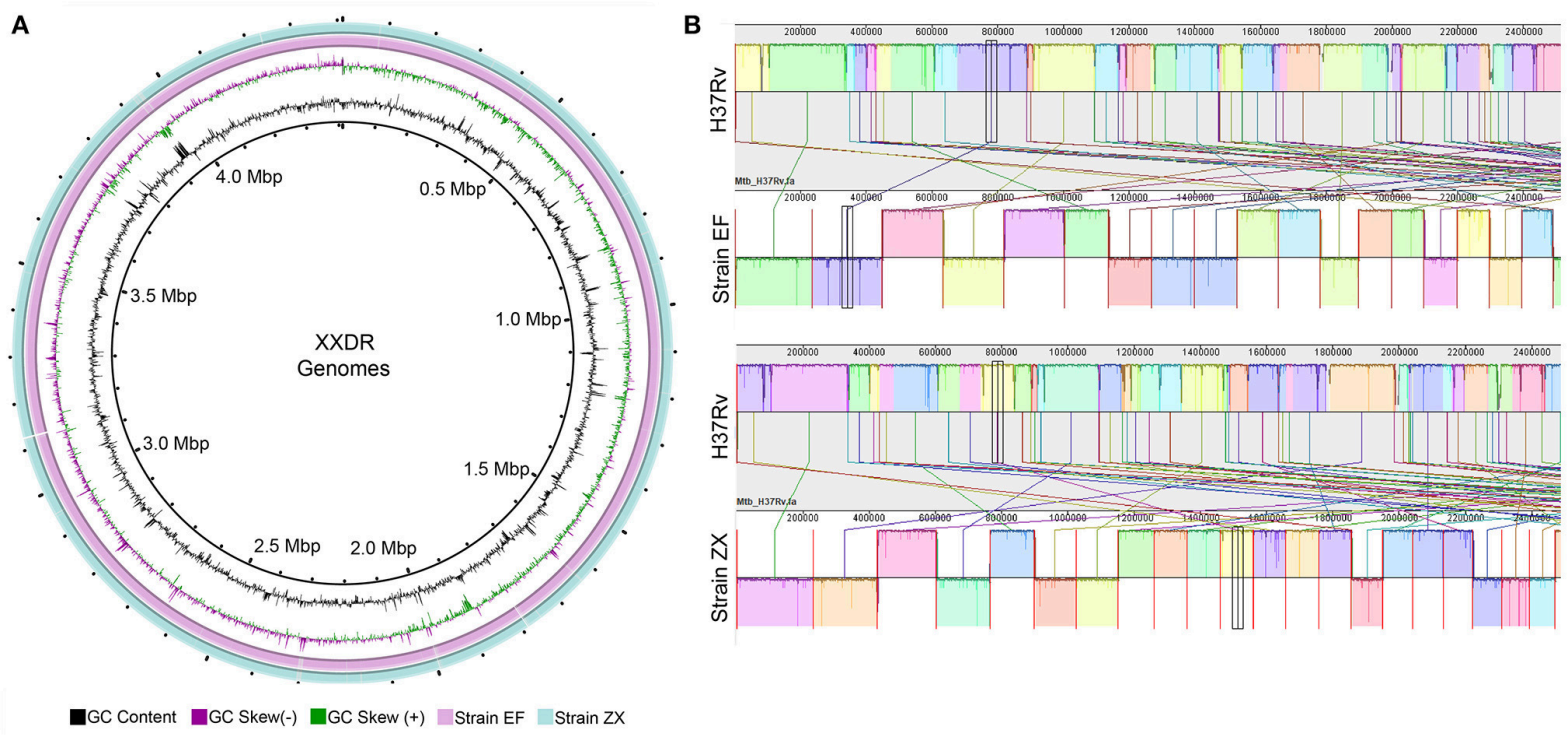
XXDR genomes and their whole genome synteny with the H37Rv reference. **(A)** Circular representation of the XXDR genome, visualized using GView. **(B)** Synteny of the two XXDR genomes with the *M. tuberculosis* H37Rv reference genome obtained using MAUVE.

The preprocessed data was *de novo* assembled using SPAdes which produced ~4.4 Mbp (Strain EF) and ~4.3 Mbp (Strain ZX) sized draft genomes. These genomes were contained in 207 and 106 contigs, respectively. The GC content of the two genomes ranged from 64 to 65%. Evaluation of the completeness and contamination levels showed that both of the genomes were >99.9% complete and showed minimal contamination. The circular representation of these two genomes is depicted in Figure 1A. We corrected the order of contigs in the draft genomes of both XXDR strains by using the *M. tuberculosis* H37Rv as a reference. The genome synteny between the XXDR genomes and the H37Rv reference is shown in Figure 1B.

### Genome Annotations

The annotation of these genomes indicated presence of 4, 210 and 4, 115 genes in strain EF and ZX, respectively. Among the genes predicted in strain EF genome, the respective number of coding sequences (CDS), transfer RNAs and tmRNAs was 4157, 52, and 1 while 4062, 52, and 1 in the ZX genome. Using RAST, a total of 1, 749 and 1, 636 genes were assigned to the subsystems in Strain EF and ZX, respectively. The RAST subsystems and the respective number of genes in both strains is as follows: Amino acids and derivatives (313, 287); carbohydrates (211, 203); cofactors, vitamins, prosthetic groups, pigments (193, 174); protein metabolism (186, 182); fatty acids, lipids, and isoprenoids (160 each); regulation and cell signaling (104, 103); DNA metabolism (96, 83); respiration (80, 74); nucleosides and nucleotides (72, 69); phosphorus metabolism (53, 47); RNA metabolism (47, 43); virulence, disease and defense (44, 41); cell wall and capsule (39, 36); membrane transport (39, 35); nitrogen metabolism (31, 24); stress response (26 each); miscellaneous (23 each); sulfur metabolism (12 each); potassium metabolism (7 each); metabolism of aromatic compounds (6 each); phages, prophages, transposable elements, plasmids (2 each); iron acquisition and metabolism (2 each); dormancy and sporulation (2 each); and secondary metabolism (1 each).

Using COG annotations, 3, 291 and 3, 225 genes were assigned function in Strain EF and ZX, respectively. These annotated genes were classified into 20 categories including lipid transport and metabolism (260, 255 genes in Strains EF and ZX respectively); transcription (233, 229); energy production and conversion (223, 217); secondary metabolites biosynthesis, transport and catabolism (220, 216); amino acid transport and metabolism (210, 204); coenzyme transport and metabolism (168, 165); replication, recombination and repair (163, 160); translation, ribosomal structure and biogenesis (162, 161); carbohydrate transport and metabolism (144, 143); inorganic ion transport and metabolism (132, 129); cell wall/membrane/envelope biogenesis (123, 119); signal transduction mechanisms (119, 116); posttranslational modification, protein turnover, chaperons (110, 109); nucleotide transport and metabolism (77, 75); cell motility (70, 69); cell cycle control, cell division, chromosome partitioning (46 each); defense mechanisms (41 each); intracellular trafficking, secretion, and vesicular transport (27, 28); RNA processing and modification (3, 2); chromatin structure and dynamics (1 each); and function unknown (759, 740). In both of genomes, 1, 653 and 1, 642 genes were annotated using Pfam whereas 1, 333 and 1, 329 genes were successfully annotated using TIGRfam database, respectively. Furthermore, the results from CRISPRfinder indicated the presence of 1 CRISPR in both of the genomes. A Venn diagram representing the overlap of functional categories (COG, Pfam, TIGRfam) is shown in Supplementary Figure 2.

### Phylogeny and Comparative Genome Analysis

**Figure 2:**
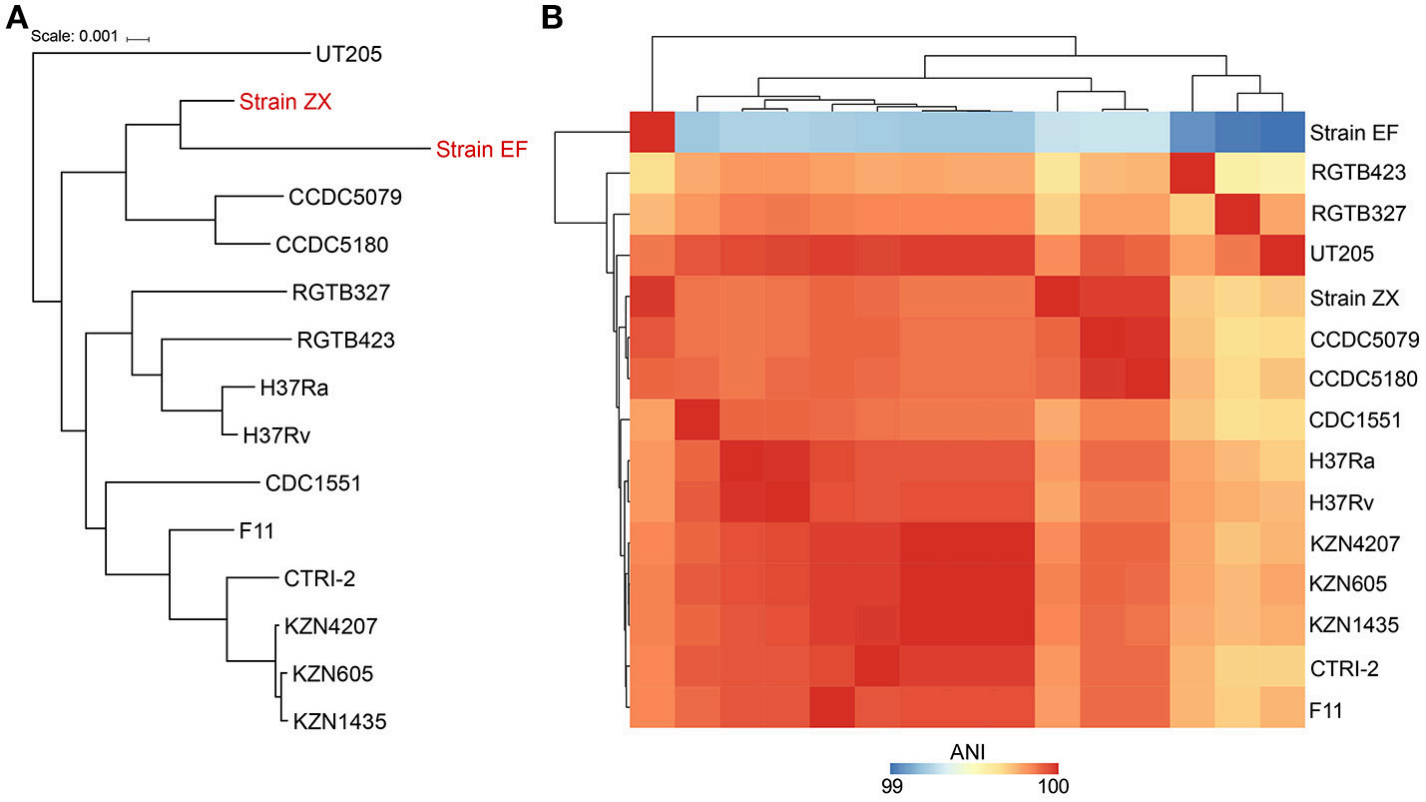
Phylogenetic classification and average nucleotide identity (ANI) of the XXDR strains. **(A)** Phylogenetic classification of the XXDR and 13 *M. tuberculosis* reference genomes. The Neighbor-joining (NJ) was constructed using the whole genome alignments and visualized using iTOL. **(B)** Average nucleotide identity (ANI) of the 15 genomes, analyzed using JSpeciesWS, the heatmap was created using R package (pheatmap).

The phylogeny was resolved by two different approaches i.e., using the 16S rRNA gene and whole genome alignments. The results from 16S rRNA gene analysis indicated that Strain ZX was grouped with strain KZN605 in the main cluster whereas Strain EF was not clustered with the other *M. tuberculosis* strains (Supplementary Figure 1). However, the phylogeny reconstructed using the whole genome alignments was better resolved as the two XXDR isolates were clustered together with *M. tuberculosis* Beijing family isolates i.e., strains CCDC5079 and CCDC5180, which are sensitive and resistant to four first line drugs, respectively (Figure 2A). ANI analysis also indicated that the two XXDR strains were highly similar to the reference genomes used in the ANI analysis as indicated by ANI of >99%. Strain ZX was almost identical to the drug resistant isolate CCDC5180 whereas Strain EF showed relatively lower similarity to the same isolate (Figure 2B and Supplementary Table 3).

### Pan-Genome and Intergenic Regions Analysis

The pan-genome analysis showed presence of 3,736 core genes shared by all of the *M. tuberculosis* genomes analyzed in this study. Furthermore, 87 soft-core genes, 115 shell genes and 506 cloud genes were also present. The number of genes specific to the Strain EF and Strain ZX was 137 and 15, respectively (Supplementary Figure 3). A phylogenetic tree of 147 strains, constructed on the basis of core gene alignments is shown in Supplementary Figure 4. The majority of unique genes in both of the strains were hypothetical proteins. However, in Strain EF, several unique genes were involved in cellular transport, metabolism and stress responses (Supplementary Table 4). In addition, pan-genome analysis also identified 29 genes which were only present in Strain EF and ZX genomes while missing in the other 145 genomes. However, the functional annotation indicated that all of these genes were hypothetical proteins. The identification of intergenic regions (IGRs), upstream of the genes, indicated presence of 2,309 and 2,286 IGRs in Strain EF and ZX, respectively. 26 IGRs were present in both of these strains, whereas 37 and 16 IGRs were specific only to Strain EF and ZX, respectively. IGRs are often associated with the differences in expression of genes and alteration of phenotype in microbes, hence they can provide key insights into the microbiology of the XXDR strains by performing functional studies.

### AR Mutation and VF Identification

Identification of causative mutations for antibiotic resistance using ResFinder indicated mutations in *katG* (S315T), *pncA* (Q10P), *rpsL* (K43R), *rpoB* (S450L) and *embB* (M360V) genes in both XXDR strains. These mutations render resistance against isoniazid, pyrazinamide, streptomycin, rifampicin and ethambutol, respectively. Furthermore, Strain ZX also showed mutations in *rrs* (A1401G), *thyA* (H75N), *gyrA* (A90V) and *gyrB* (A504V) genes which result in resistance against amikacin, PAS and fluoroquinolones, respectively. We also identified virulence factors in both of the genomes which were associated with the categories of secretion system type VII, iron uptake, cellular metabolism, iron uptake, and heat-shock protein etc. Most noticeable examples of VFs in these two genomes included secreted proteins (*espBC*), membrane proteins (*eccB1, eccB3, eccB5, eccE1* etc.), esterase, lipase, polyketide synthetase (*mbtD*), and iron dependent repressor (*ideR*/*dtxR*).

In the current study, we *de novo* assembled draft genomes of the two XXDR strains identified from Beijing and also performed their functional annotations. These genome sequences could serve as a useful reference for performing comparative genomics and evolutionary studies in the near future to increase the understanding of mechanisms of drug resistance in *M. tuberculosis*. Furthermore, in contrast with the initial report (Li et al., 2015), we identified H75N mutation in the genome sequence which is an indicative of the influence of the workflow used for the data analysis on the outcomes. Therefore, for using sequencing in diagnostics, special attention should be paid to the data analysis. Furthermore, the unique genes, IGRs and the virulence factors identified from annotations can play key role in designing novel therapies to better control XXDR TB.

## Author Contributions

MRK and Y-CZ performed the genomic assembly, annotation, comparative genome and pangenome analyses and wrote the manuscript. F-CX, KK, and H-YL performed the antibiotic resistance and virulence factor identification. H-TZ edited the manuscript and supervised the study. All authors have read and approved the manuscript.

### Conflict of Interest Statement

The authors declare that the research was conducted in the absence of any commercial or financial relationships that could be construed as a potential conflict of interest.
