# Supplementary material for: Genome Sequences and Comparative Analysis of Two Extended-Spectrum Extensively-Drug Resistant Mycobacterium tuberculosis Strains
Source: Front Pharmacol. 2018 Dec 18;9:1492. doi: 10.3389/fphar.2018.01492 (PMC6305476; doi:10.3389/fphar.2018.01492)
Supplement: Supplementary file 6 [file Image_2.pdf]

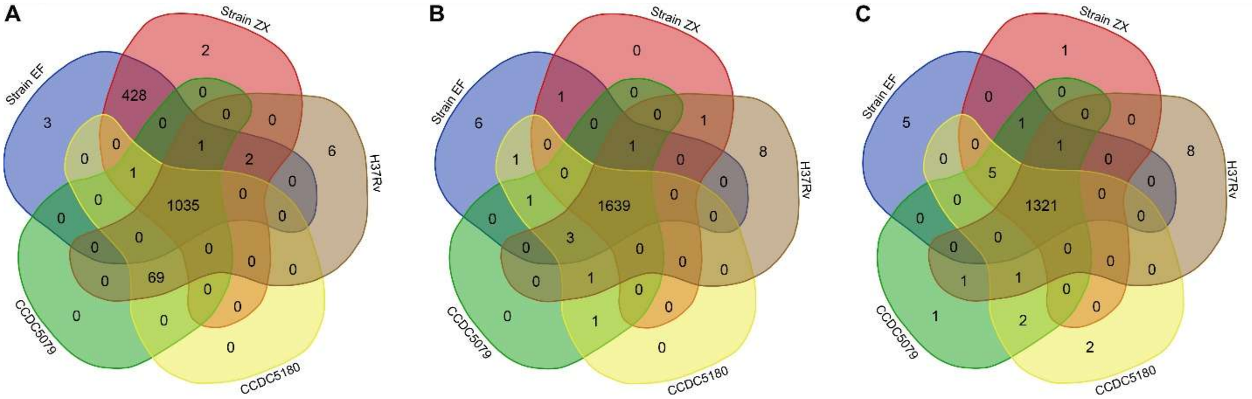

Supplementary Figure 2 | Venn diagram representing the number of functional categories shared between the five *M. tuberculosis* strains. **(A)** COG, **(B)** Pfam and **(C)** TIGRfam. We only compared the functional categories shared among the genomes of Strain EF and ZX with the *M. tuberculosis* H37Rv, *M. tuberculosis* CCDC5079 and *M. tuberculosis* CCDC5180 strains.
