# Supplementary material for: Genome Sequences and Comparative Analysis of Two Extended-Spectrum Extensively-Drug Resistant Mycobacterium tuberculosis Strains
Source: Front Pharmacol. 2018 Dec 18;9:1492. doi: 10.3389/fphar.2018.01492 (PMC6305476; doi:10.3389/fphar.2018.01492)
Supplement: Supplementary file 8 [file Image_4.pdf]

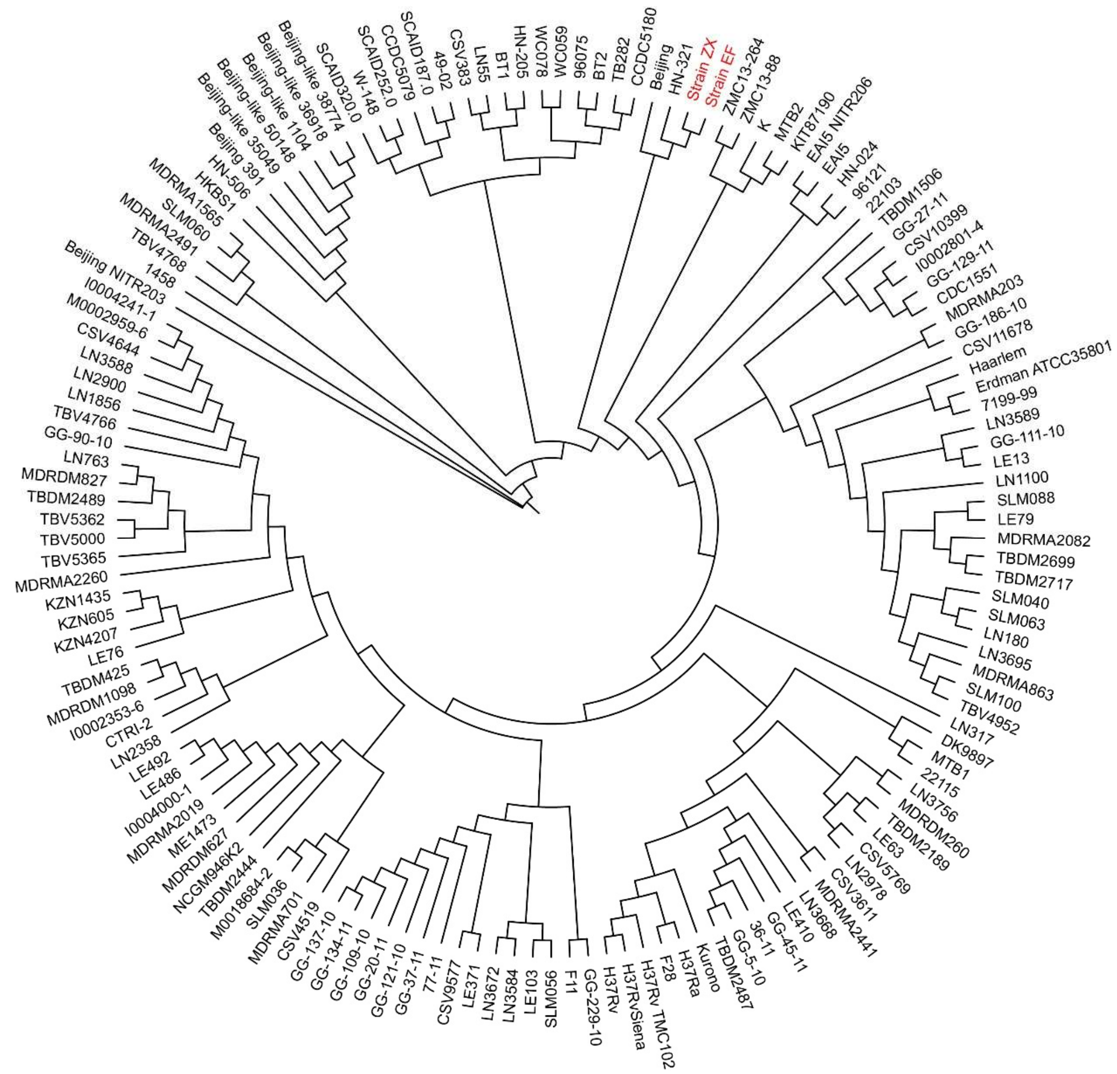

Supplementary Figure 4 | Phylogenetic tree based on the core gene alignments from the 147 *M. tuberculosis* genomes.
